# Development and analytical validation of a multiplex qPCR assay for *Toxoplasma gondii*, *Treponema pallidum*, cytomegalovirus, and herpes simplex virus types 1 and 2 in neonates

**DOI:** 10.7717/peerj.21695

**Published:** 2026-09-15

**Authors:** Abdirahman Hussein Elmi, Chan Yean Yean, Nor Rosidah Ibrahim, Manal Abdel Haleem A. Abusalah, Zeehaida Mohamed

**Affiliations:** 1 Department of Medical Microbiology and Parasitology, School of Medical Sciences, Health Campus, Universiti Sains Malaysia, Kubang Kerian, Malaysia; 2 Department of Medical Laboratory Sciences, Faculty of Medicine and Health Sciences, Jamhuriya University of Science and Technology, Mogadishu, Somalia; 3 Department of Pediatrics, School of Medical Sciences, Health Campus, Universiti Sains Malaysia, Kubang Kerian, Malaysia; 4 Hospital Pakar Universiti Sains Malaysia, Kubang Kerian, Malaysia

**Keywords:** Multiplex qPCR, Congenital infections, Neonatal screening, Molecular diagnostics, *Toxoplasma gondii*, *Treponema pallidum*, CMV, HSV

## Abstract

**Background:**

Congenital and perinatal infections caused by pathogens such as *Toxoplasma gondii* (*T. gondii*), *Treponema pallidum* (*T. pallidum*), cytomegalovirus (CMV), and herpes simplex virus types 1 and 2 (HSV-1 and HSV-2) are significant causes of neonatal morbidity. Traditional diagnostic methods, such as serological tests or viral cultures, are frequently limited by cross-reactivity, variable accuracy, and prolonged turnaround times. Therefore, this study aimed to design and analytically validate a rapid, highly sensitive, and specific multiplex quantitative real-time polymerase chain reaction (qPCR) assay for the detection of these critical neonatal pathogens.

**Methods:**

The multiplex qPCR assay was optimized, and analytical performance, including sensitivity, specificity, and efficiency, was evaluated following Minimum Information for Publication of Quantitative Real-Time PCR Experiments (MIQE) guidelines.

**Results:**

The assay demonstrated a limit of detection (LOD) of 10 attograms per microliter (ag/µL) for *T. gondii, T. pallidum*, and CMV, and 1 ag/µL for herpes simplex virus (HSV)-1 and HSV-2. Additionally, the assay showed 100% analytical specificity against 38 organisms. The PCR efficiency exhibited ranged between 95% and 109% for each organism, with a coefficient of variation (CV) of less than 3%.

**Conclusion:**

This multiplex qPCR assay provides highly sensitive and specific analytical performance for detecting key congenital pathogens. It represents a promising high-throughput tool with the potential to improve the turnaround time of neonatal molecular screening.

## Introduction

Congenital and perinatal infections are caused by a specific group of maternal pathogens, including *Toxoplasma gondii* (*T. gondii*), *Treponema pallidum* (*T. pallidum*), cytomegalovirus (CMV), and herpes simplex virus (HSV) (Nahmias et al., 1971; Chaudhary et al., 2024; Kahraman Kilbas et al., 2025). While maternal infection is often asymptomatic, placental transmission carries a high risk of severe fetal complications, including miscarriage, stillbirth, and intrauterine growth restriction (Chung, Shin & Lee, 2018; Chatzakis et al., 2020; DeRose et al., 2023).

Prenatal screening is not available in numerous low- and middle-income countries for the early identification of these congenital infections (Menendez et al., 2017). Moreover, clinical presentations are often misinterpreted, as these infections frequently mimic neonatal sepsis or other conditions (Morsy, Hussein & Morsy, 2022).

Serological testing for neonates can be problematic because maternal IgG antibodies may persist in the infant’s bloodstream for an extended duration, complicating the assessment of actual infection status. Conversely, IgM antibodies are frequently inconsistent and prone to cross-reactivity. In addition, pathogen culture requires a long time and delays treatment (Dolgushin et al., 2015; Chung, Shin & Lee, 2018; Batra, Batra & Singh, 2020).

Molecular techniques, especially quantitative real-time polymerase chain reaction (qPCR), are quick, sensitive, and specific in detecting the pathogen’s DNA (Yamamoto et al., 2020). However, neonates have limited sample volume, and testing for each pathogen separately needs an excessive amount of blood or cerebrospinal fluid (Russcher et al., 2020).

To address this, multiplex qPCR allows for the detection of several pathogens in a single reaction. Ultimately, such a molecular tool aims to provide a fast and cost-effective way to improve the clinical care and outcomes of newborns suspected of having congenital infections. Therefore, the goal of this study was to develop and validate a reliable multiplex qPCR test for detecting *T. gondii, T. pallidum*, CMV, herpes simplex virus-1 (HSV-1), and herpes simplex virus-2 (HSV-2).

## Materials and Methods

### Oligonucleotide design

Primers and probes of specific target pathogens were designed using the IDT DNA PrimerQuest online tool (https://sg.idtdna.com/PrimerQuest/) based on conserved gene sequences with GenBank accession numbers retrieved from the NCBI database: *T. gondii* (*B1* gene, AF179871.1), *T. pallidum* (*tpp47* gene, M88769.1), CMV (*MIE* gene, M21295.1), HSV-1 (*gD* gene, K02372), and HSV-2 (*gB* gene, M14923.1). The specificity of all candidate oligonucleotide sequences was verified *in silico via* Primer-BLAST analysis (https://blast.ncbi.nlm.nih.gov/Blast.cgi). To facilitate assay development, synthetic double-stranded DNA standards (gBlocks Gene Fragments) were custom-designed to serve as positive controls using the IDT DNA PrimerQuest online tool (https://sg.idtdna.com/pages/products/genes/gblocks-gene-fragments/).

Furthermore, potential primer-dimer and hairpin interactions were evaluated, specifically screening for homodimer and heterodimer structures with Gibbs free energy (ΔG) values indicating high stability (ΔG≤ −9 kcal/mol).

### Quantitative real-time PCR assay setup

Quantitative analysis was performed using the Bio-Rad CFX96 Touch Real-Time PCR System, with data acquisition and analysis managed *via* the Bio-Rad CFX Manager Software (Bio-Rad, Hercules, CA, USA). The multiplex assay was organized into two sets: SET 1 targeted CMV, HSV-1, and HSV-2, while SET 2 focused on *T. gondii* and *T. pallidum*. In addition we included an internal amplification control (IAC) to ensure the reliability of the results (Ali, 2018). Each reaction contained 10 µL of 1X modified KiCqStart One-Step Probe RT-qPCR master mix. The optimized concentrations for SET 1 were 0.9 µM for CMV primers and probe (FAM), 0.5 µM for HSV-1 and HSV-2 primers and 0.2 µM for HSV-1 (Cy5.5) and HSV-2 (Texas Red) probes. For SET 2, we used concentrations of 0.7 µM for the *T. gondii* primer and probe (FAM) and 0.9 µM for the *T. pallidum* primer and probe (Cy5.5). Additionally, the IAC primer concentration was 0.5 µM and the IAC probe (Cy5) was 0.2 µM. For each reaction, 2 µL of the target template at 20 femtograms per microliter (fg/µL) and 0.5 µL of the IAC (2 fg/µL) were added to each well, bringing the final reaction volume to 20 µL. All reagents were stored at −20 °C. Furthermore, the thermal cycling protocol started with an initial denaturation at 95 °C for 3 min, followed by 40 cycles of 95 °C denaturation for 10 s and 60 °C annealing and extension for 30 s.

### PCR efficiency, linearity, and intra-assay variability

PCR efficiency (E) was calculated using the formula E = 10ˆ^(−1/slope)^ –1, and linearity was determined by the coefficient of determination (R^2^) of the calibration curves. The standard curves were generated by plotting the log template concentrations on the *X*-axis against the mean Cq values on the *Y*-axis, using a 6-point, 10-fold serial dilution series (100 fg/µL, 10 fg/µL, 1 fg/µL, 100 ag/µL, 10 ag/µL, and 1 ag/µL) tested in triplicate. To measure intra-assay variability, we calculated the coefficient of variation (CV%) from triplicate results in a single run at two specific concentration points (100 fg/µL and 10 ag/µL).

### Analytical sensitivity and specificity

Analytical sensitivity was defined as the limit of detection (LOD), which is the lowest DNA concentration detectable in all triplicates or with 95% confidence (Winters et al., 2022; Truong & Šlapeta, 2023). We tested 10-fold serial dilutions at 100 fg/µL, 10 fg/µL, 1 fg/µL, 100 ag/µL, 10 ag/µL, and 1 ag/µL. The copy number conversions were obtained using the Thermo Scientific online calculator based on a uniform 500 bp gBlock fragment length used as the template standard for all five organisms.

Finally, we assessed analytical specificity using a panel of 38 genomic DNA samples from various bacteria, fungi, and viruses to ensure no cross-reactivity occurred.

## Results

### Oligonucleotide design

The primers and probes were successfully designed using the IDT DNA PrimerQuest tool, ensuring that probes were positioned between the forward and reverse primers. *In silico* analysis through Primer-BLAST confirmed the high specificity of all sequences, which showed 100% exclusivity against the NCBI Nucleotide database. No cross-reactivity with human genomic DNA or other micro-organisms was detected.

The designed oligonucleotides featured primer lengths between 20 and 22 bp, with GC contents ranging from 48% to 55% and melting temperatures (Tm) of 61–64 °C. Probes ranged from 24 bp to 25 bp in length, with a Tm approximately 5–10 °C higher than the primers (Table 1).

To avoid assay inhibition, secondary structures were screened. Three primers showed ΔG values ranging from −9.28 to −12.9 kcal/mol in Supplemental File S1 (Table S1A), and two probes exhibited self-dimers with ΔG values between −9.75 and −13.8 kcal/mol (Table S1B). In addition, one forward primer-probe interaction showed a ΔG value of −10.09 kcal/mol (Table S1C).

### Quantitative multiplex qPCR optimization and performance

For optimization results in SET 1, CMV amplification yielded a Cq of 23.29 with a relative fluorescence unit (RFU) of 944. Meanwhile, HSV-1 and HSV-2 produced high RFU values of 1,926 and 1,099, respectively. In SET 2, *T. gondii* and *T. pallidum* achieved minimum RFU values of 1,140 and 1,195. Furthermore, the IAC showed consistent amplification both sets with a Cq range of 26.53 to 27.11. All negative controls (NC) remained undetected (File S2). Additionally, the baseline thresholds were set at 25 for all channels, except for Cy5.5, which was set at 50; a Cq value ≤ 37 was used to define a positive result.

### Linearity and PCR efficiency

**Figure 1:**
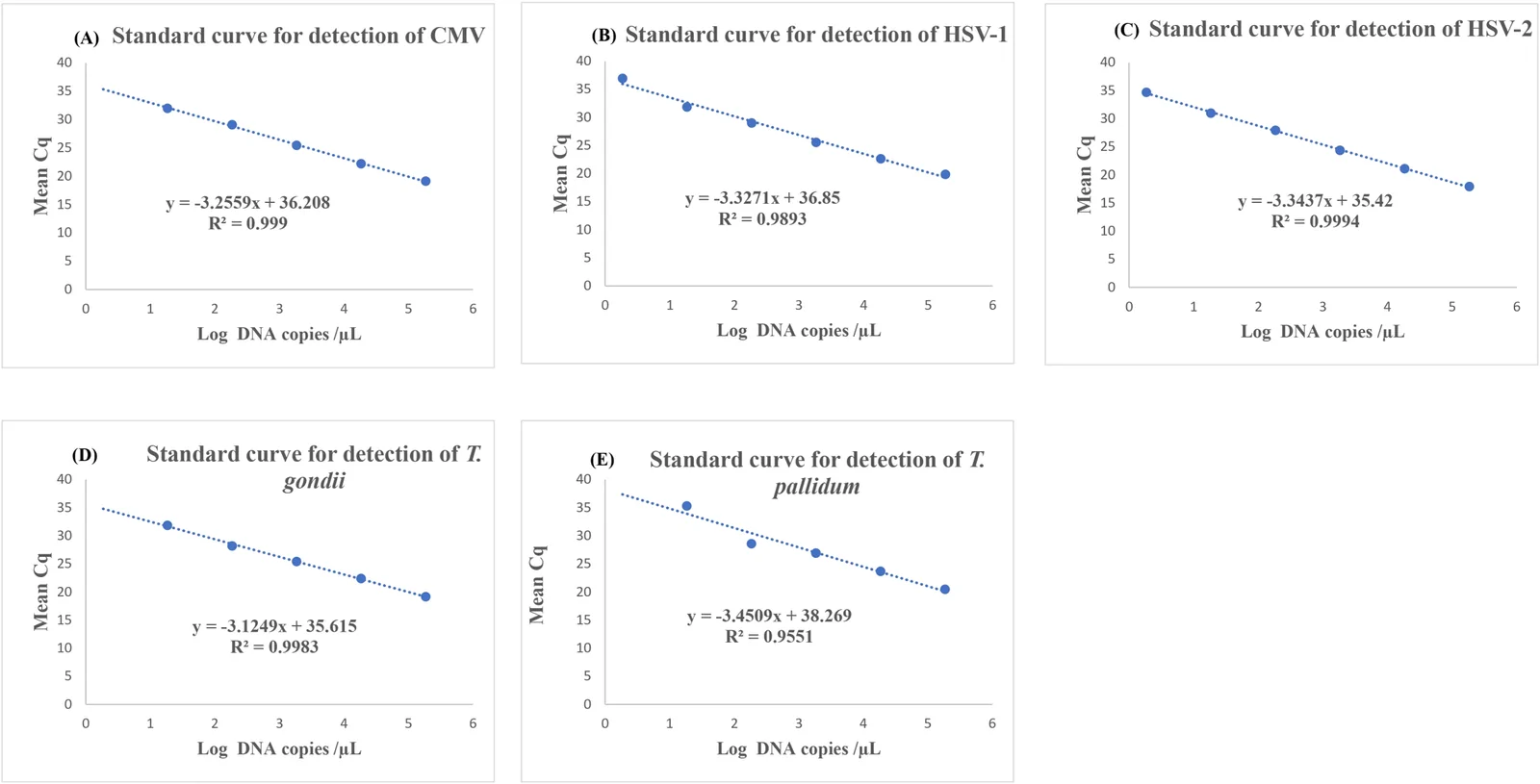
Standard curve of the developed qPCR for the detection of (A) CMV, (B) HSV-1, (C) HSV-2. (D) *T. gondii* and (E) *T. pallidum*.

For SET 1, amplification efficiencies were 103% (CMV), 100% (HSV-1), and 99% (HSV-2), while SET 2, efficiencies were 109% (*T. gondii*) and 95% (*T. pallidum*). All results are showing in Figs. 1A–1E. In addition, the linearity (R^2^) values generally exceeded 0.98 for most targets. Although *T. pallidum* showed a slightly lower linearity (R^2^ = 0.955). The slope values for all five pathogens fell within the optimal range of −3.12 to −3.45 (Table 2).

**Table 1:** Characteristics of primers and probes designed for this study.

**Organism**	**Nucleotide sequence (5′–3′)**	**Amplicon length (bp)**	**Length (bp)**	**Tm (**°**C)**	**GC (%)**
*T. gondii*	F-TACGACATCGCATTCAAGGG	157	20	62	50
	FAM-CCCACAAGACGGCTGAAGAATGCAA-BHQ_1		25	69	52
	R-GTTATGATGCAACACGTACCC		21	61	48
*T. pallidum*	F-ATCTGATGGCGAGTTATGGC	170	20	62	50
	Cy5.5-AAAGGTTCAGGGCCGGGATACTACA-BHQ_2		25	69	52
	R-AGCAACTACGTCCCTATACCC		21	63	52
CMV	F-AGGATAAGCGGGAGATGTGG	184	20	63	55
	FAM-AAGGAGCTGCATGATGTGAGCAAGG-BHQ_1		25	69	52
	R-GGGAAGGCTGAGTTCTTGGTA		21	64	52
HSV-1	F-AAGACGTCCGGAAACAACCC	122	20	64	55
	Cy5.5-TTCGGATGGGAGGCAACTGTGCTA-BHQ_2		24	69	54
	R-GACTTGTTGTAGGAGCATTCGG		22	63	50
HSV-2	F-ACACAAGGCCAGAAAGAAGG	135	20	62	50
	Texas Red-AGGTCACCAACATGGTTCTGCGCAA-BHQ_2		25	70	52
	R-TAGAGCTCGTCTTCGTCTCC		20	62	55

**Notes.**

F forword R Reverse

**Table 2:** Performance characteristics of the developed multiplex qPCR assays.

**Organism**	**Efficiency (%)**	**Linearity (R^2^)**	**Slope**
CMV	103	0.9990	−3.2559
HSV-1	100	0.9893	−3.3271
HSV-2	99	0.9994	−3.3437
*T. gondii*	109	0.9983	−3.1249
*T. pallidum*	95	0.9551	−3.4509

### Intra-assay variability

The assay also demonstrated high precision, with intra-assay variability (CV%) ranging from 0.10% to 2.89%. At high template concentrations (100 fg/µL) variability was consistently below 2%, while at low concentration (10 ag/µL), it remained low, with only CMV reaching a CV% of 2.89% as shown in Table 3.

**Table 3:** Intra-assay variability of the multiplex real-time PCR (*n* = 3).

**Organism**	**DNA Amount**	**Mean Cq**	**SD**	**CV (%)**
CMV	100 fg	19.12	0.36	1.79
	10 ag	31.97	0.91	2.89
HSV-1	100 fg	19.86	0.22	1.12
	10 ag	31.85	0.44	1.37
HSV-2	100 fg	17.93	0.16	0.89
	10 ag	30.99	0.19	0.62
*T. gondii*	100 fg	19.15	0.15	0.77
	10 ag	31.87	0.42	1.31
*T. pallidum*	100 fg	20.48	0.02	0.10
	10 ag	35.29	0.36	1.02

### Analytical sensitivity and specificity

**Figure 2:**
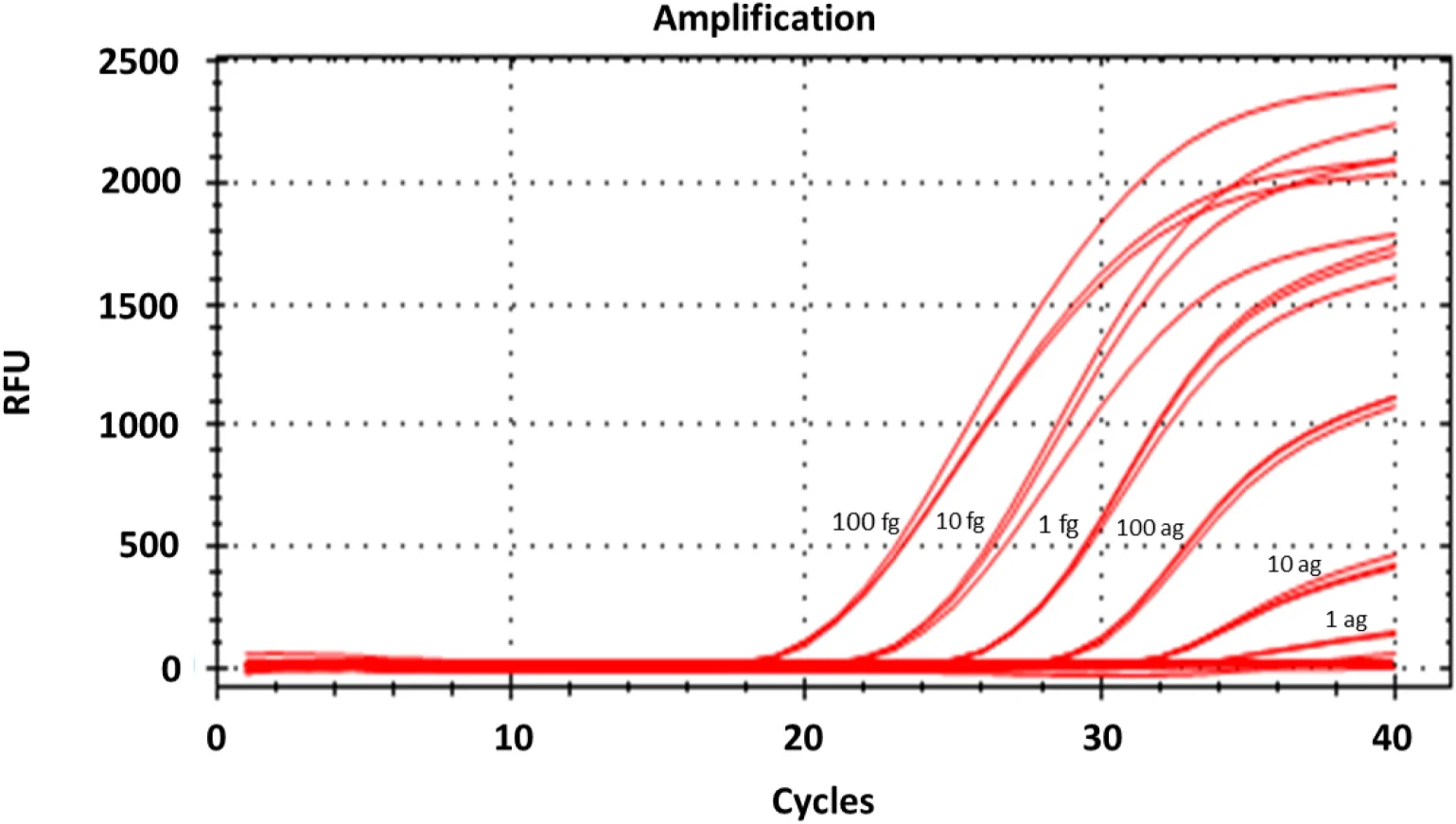
Representation of multiplex qPCR amplification curves for LOD. The plot shows RFU *versus* PCR cycle number for a range of DNA concentrations.

The assay was highly sensitive with an LOD of 10 ag/µL (18.5 copies/µL) for CMV, *T. gondii*, and *T. pallidum*. Notably, the assay showed even greater sensitivity for HSV-1 and HSV-2, reaching an LOD of 1 ag/µL (1.85 copies/µL). The results are summarized in Table 4 and a representative amplification plot is shown in Fig. 2.

**Table 4:** Analytical sensitivity (LOD) results for multiplex qPCR targets.

**Organism**	**DNA amount**	**DNA copies/µL**	**Log DNA copies /µL**	**Detection rate**	**Mean Cq**	**SD**
CMV	100 fg	185,292.3	5.268	3/3	19.12	0.36
	10 fg	18,529.23	4.268	3/3	22.22	0.58
	1 fg	1,852.92	3.268	3/3	25.45	0.39
	100 ag	185.29	2.268	3/3	29.08	0.25
	10 ag	18.52	1.268	3/3	31.97	1.97
	1 ag	1.853	0.268	0/3	ND	ND
HSV-1	100 fg	185,292.3	5.268	3/3	19.86	0.22
	10 fg	18,529.23	4.268	3/3	22.63	0.17
	1 fg	1,852.92	3.268	3/3	25.57	0.21
	100 ag	185.29	2.268	3/3	29.01	0.04
	10 ag	18.52	1.268	3/3	31.85	0.44
	1 ag	1.853	0.268	2/3	36.93	0.16
HSV-2	100 fg	185,292.3	5.268	3/3	17.93	0.16
	10 fg	18,529.23	4.268	3/3	21.09	0.04
	1 fg	1,852.92	3.268	3/3	24.36	0.02
	100 ag	185.29	2.268	3/3	27.94	0.10
	10 ag	18.52	1.268	3/3	30.99	0.19
	1 ag	1.853	0.268	3/3	34.68	2.17
*T. gondii*	100 fg	185,292.3	5.268	3/3	19.15	0.15
	10 fg	18,529.23	4.268	3/3	22.39	0.09
	1 fg	1,852.92	3.268	3/3	25.41	0.21
	100 ag	185.29	2.268	3/3	28.20	0.11
	10 ag	18.52	1.268	3/3	31.87	0.42
	1 ag	1.853	0.268	0/3	ND	ND
*T. pallidum*	100 fg	185,292.3	5.268	3/3	20.48	0.02
	10 fg	18,529.23	4.268	3/3	23.69	0.16
	1 fg	1,852.92	3.268	3/3	26.92	0.23
	100 ag	185.29	2.268	3/3	28.58	0.39
	10 ag	18.52	1.268	3/3	35.29	0.36
	1 ag	1.853	0.268	0/3	ND	ND

**Notes.**

ND Not Detected

According to the analytical specificity, the assay demonstrated 100% specificity against a panel of 38 non-target microorganisms including bacteria, fungi, and viruses. No cross-reactivity or false positive was detected in the pathogen-specific channels. Importantly the IAC was successfully amplified in all reactions, confirming the validity of negative results. The panel of 38 genomic DNA samples used in this study is detailed in File S3.

## Discussion

In this study, we successfully developed a multiplex qPCR assay for the detection of *T. gondii*, *T. pallidum*, CMV, HSV-1, and HSV-2. Unlike traditional serology, which is often limited by maternal antibody interference and cross-reactivity (Zimmerman, Bonon & Marba, 2025), this molecular approach offers a rapid and precise alternative. This study provides a rapid multiplex qPCR with high analytical sensitivity and specificity.

In the oligonucleotide design, for *in silico* investigation, we identified some thermodynamic issues; particularly, three primers and two probe interactions showed ΔG values ranging from −9.75 to −13.8 kcal/mol. In primer and probe with ΔG value below −9 kcal/mol is often used as a threshold for heterodimers or self-dimers (Asif et al., 2021; Ismadi, Mohamad & Harun, 2024). The thermodynamic predictions are helpful, while final reaction parameters like annealing temperature and primer concentration play a more influential role in success (Ismadi, Mohamad & Harun, 2024).

Furthermore, during optimization, we incorporated an IAC to detect potential PCR issues like PCR inhibition (Moldovan & Moldovan, 2020; Ismadi, Mohamad & Harun, 2024). The IAC was selected for its high selectivity and a sufficient copy number to improve the assay’s sensitivity, thereby facilitating dependable detection at low target concentrations (Ali, 2018).

For PCR efficiency our results showed high efficiency with an ideal range between 90% and 110% (Bustin et al., 2009). Our result is higher than the values reported by Gupta, Lawrence & Fraley (2023) which were 85% for HSV-1, 58% for HSV-2, and 77% for CMV. But it aligns with a study done by Sahoo et al. (2022) who reported 96% for CMV. For *T. gondii*, the efficiency result was lower than the study conducted by Berizi et al. (2021) reported at 113%, meanwhile *T. pallidum* demonstrated efficiency at 95%, agrees with the 93% efficiency reported by Rajendiran et al. (2021). Regarding to the linearity, the coefficient of determination (R^2^) exceeded 0.98 for all pathogens except for *T. pallidum* which showed R^2^ of 0.955 below the ideal range (Bustin et al., 2009), indicating a strong correlation between template concentration and Cq value. According to the slope of the standard curve for all pathogens remained within the optimal range (Bustin et al., 2009), confirming that the amplification kinetics were efficient and reproducible despite the minor linearity deviation (Jiang et al., 2022).

The limit detection remained consistent at 10 ag/µL (18.5 copies/µL) for *T. gondii*, *T. pallidum*, and CMV. This result aligns with previous studies that reported a LOD of 10 ag (Zhou et al., 2015; Suviriyapaisal, Wanachiwanawin & Limawongpranee, 2017; Li et al., 2023; Xu et al., 2024). Additionally, the HSV-1 and HSV-2 showed an LOD of 1 ag/µL (1.8 copies/µL). This is crucial for the early identification and rapid treatment of congenital infections. Previous studies reported an LOD 35 copies/µL and 90 to 200 copies/mL (Pillet et al., 2015; Gupta, Lawrence & Fraley, 2023).

Finally, the analytical specificity showed no amplification signals against the 38 organisms tested, and the IAC amplified successfully in all channels, indicating a lack of PCR inhibition. Analytical specificity is essential for any diagnostic assay, as any cross-reactivity with other pathogens may lead to misdiagnosis and inappropriate treatment (Bustin et al., 2009; Smith, 2021).

In this study, the analytical specificity panel was limited to the organisms available in our laboratory collection. To better assess the assay’s exclusivity, future validation should include a wider range of viral and protozoan species. Furthermore, this study primarily focused on analytical performance using synthetic standards. Future research should include a full clinical evaluation using true patient specimens such as blood, cerebrospinal fluid, urine, saliva and amniotic fluid to confirm how the assay performs in a diagnostic setting where factors like low parasitic loads and PCR inhibitors can affect sensitivity.

## Conclusions

The current study developed a highly analytically sensitive and specific multiplex qPCR assay capable of detecting five congenital pathogens. By resolving the complexities of oligonucleotide design, thermodynamic risks, and fluorophore interference through optimization, we designed a diagnostic tool that maintains high performance standards, demonstrating PCR efficiencies from 95% to 109% and a limit of detection of 1 ag/µL for HSV-1 and HSV-2, and 10 ag/µL for CMV, *T. gondii*, and *T. pallidum*. Once clinically validated, using this assay in clinical laboratories could markedly enhance the speed and precision of identifying congenital infections, facilitating earlier treatment.

##  Supplemental Information

10.7717/peerj.21695/supp-1 Supplemental Information 1 MIQE checklist

10.7717/peerj.21695/supp-2 Supplemental Information 2 Raw data for qPCR validation and standard curves of target pathogens The raw experimental quantitative polymerase chain reaction (qPCR) replication profiles across a 6-point serial 10-fold dilution range (100 femtograms per microliter down to 1 attogram per microliter) for five panel pathogens.

10.7717/peerj.21695/supp-3 Supplemental Information 3 Self Dimer and Hetero Dimer

10.7717/peerj.21695/supp-4 Supplemental Information 4 Performance of the optimized Multiplex assay

10.7717/peerj.21695/supp-5 Supplemental Information 5 Analytical specificity results against non-target microorganisms

## Additional Information and Declarations

### Data Availability

The following information was supplied regarding data availability: The data is available in the Supplemental Files.

## References

[ref-1] Ali MRBM (2018). Development of thermostable multiplex qPCR for simultaneous detection of pathogens associated with febrile illnesses. PhD Thesis.

[ref-2] Asif S, Khan M, Waqar Arshad M, Shabbir MI (2021). PCR optimization for beginners: a step by step guide. Research in Molecular Medicine.

[ref-3] Batra P, Batra M, Singh S (2020). Epidemiology of TORCH infections and understanding the serology in their diagnosis. Journal of Fetal Medicine.

[ref-4] Berizi M, Babaie J, Fard-Esfahani P, Enshaeieh M, Noordin R, Saadatnia G, Golkar M (2021). Development of a quantitative real-time pcr assay for detection of toxoplasma gondi in brain samples. Iranian Journal of Parasitology.

[ref-5] Bustin SA, Benes V, Garson JA, Hellemans J, Huggett J, Kubista M, Mueller R, Nolan T, Pfaffl MW, Shipley GL, Vandesompele J, Wittwer CT (2009). The MIQE guidelines: minimum information for publication of quantitative real-time PCR experiments. Clinical Chemistry.

[ref-6] Chatzakis C, Ville Y, Makrydimas G, Dinas K, Zavlanos A, Sotiriadis A (2020). Timing of primary maternal cytomegalovirus infection and rates of vertical transmission and fetal consequences. American Journal of Obstetrics and Gynecology.

[ref-7] Chaudhary R, Patel SS, Sinha R, Tejan N, Arya A, Sahu C (2024). Seroprevalence of TORCH infection in paediatric population and women of reproductive age group: a cross-sectional study. Indian Journal of Neonatal Medicine and Research.

[ref-8] Chung MH, Shin CO, Lee J (2018). TORCH (Toxoplasmosis, rubella, cytomegalovirus, and herpes simplex virus) screening of small for gestational age and intrauterine growth restricted neonates: efficacy study in a single institute in Korea. Korean Journal of Pediatrics.

[ref-9] DeRose DU, Bompard S, Maddaloni C, Bersani I, Martini L, Santisi A, Longo D, Dotta ARonchettiMP, Auriti C (2023). Neonatal herpes simplex virus infection: from the maternal infection to the child outcome. Journal of Medical Virology.

[ref-10] Dolgushin SA, Odintsova ES, Gerasimenko AY, Tronin AV, Tereshchenko SA (2015). Preliminary tests of multiplex immunoassay for detection of TORCH infections in human blood serum using flow cytometry. Biomedical Engineering.

[ref-11] Gupta A, Lawrence SM, Fraley SI (2023). A broad-based probe-free qPCR assay for detection and discrimination of three human herpes viruses. Journal of Virological Methods.

[ref-12] Ismadi YKM, Mohamad S, Harun A (2024). Development of multiplex real-time PCR for simultaneous detection of common fungal pathogens in invasive mycoses. PeerJ.

[ref-13] Jiang X-W, Huang T-S, Xie L, Chen S-Z, Wang S-D, Huang Z-W, Li X-Y, Ling W-P (2022). Development of a diagnostic assay by three-tube multiplex real-time PCR for simultaneous detection of nine microorganisms causing acute respiratory infections. Scientific Reports.

[ref-14] Kahraman Kilbas EP, Ciftci IH, Kilbas I, Toptan H (2025). Seroprevalence of TORCH viral agents in pregnant women in Turkey: systematic review and meta-analysis. Pathogens.

[ref-15] Li M, Lv Y, Cui D, Xu Y, Lin M, Zhang X, Wang Y, Shen C, Xie J (2023). Development and clinical validation of a one-step pentaplex real-time reverse transcription PCR assay for detection of hepatitis virus B, C, E, *Treponema pallidum*, and a human housekeeping gene. BMC Infectious Diseases.

[ref-16] Menendez C, Castillo P, Martínez MJ, Jordao D, Lovane L, Ismail MR, Carrilho C, Lorenzoni C, Fernandes F, Nhampossa T, Hurtado JC, Navarro M, Casas I, Santos RP, Bandeira S, Mocumbi S, Jaze Z, Mabota F, Munguambe Kh, Maixenchs M, Sanz A, Mandomando I, Nadal A, Goncé A, Muñoz Almagro C, Quintó L, Vila J, Macete E, Alonso P, Ordi J, Bassat Q (2017). Validity of a minimally invasive autopsy for cause of death determination in stillborn babies and neonates in Mozambique: an observational study. PLOS Medicine.

[ref-17] Moldovan E, Moldovan V (2020). Controls in real-time polymerase chain reaction based techniques. Acta Marisiensis—Seria Medica.

[ref-18] Morsy T, Hussein H, Morsy A (2022). Torch infections, pathogenicity & mortality assessments. Journal of the Egyptian Society of Parasitology.

[ref-19] Nahmias AJ, Walls KW, Stewart JA, Herrmann KL, Flynt WJ (1971). The ToRCH complex-perinatal infections associated with toxoplasma and rubella, cytomegol- and herpes simplex viruses. Pediatric Research.

[ref-20] Pillet S, Verhoeven PO, Epercieux A, Bourlet T, Pozzetto B (2015). Development and validation of a laboratory-developed multiplex real-time PCR assay on the BD max system for detection of herpes simplex virus and varicella-zoster virus DNA in various clinical specimens. Journal of Clinical Microbiology.

[ref-21] Rajendiran P, Saravanan N, Ramamurthy M, Sankar S, Aruliah R, Nandagopal B, Sridharan G (2021). Standardization of an in-house multiplex real-time polymerase chain reaction for the simultaneous detection of *Toxoplasma gondii*, Rubella virus, cytomegalovirus, herpes simplex Virus 1 and 2, and *Treponema pallidum* infection among pregnant women. Indian Journal of Public Health.

[ref-22] Russcher A, Enders A, De Brouwer CS, Oepkes D, Hahn R, Enders M, Kroes ACM, Vossen ACTM (2020). Diagnosis of intrauterine parvovirus B19 infection at birth –Value of DNA detection in neonatal blood and dried blood spots. Journal of Clinical Virology.

[ref-23] Sahoo S, Mandal S, Das P, Bhattacharya S, Chandy M (2022). An analysis of the standard curve parameters of cytomegalovirus, BK virus and hepatitis B virus quantitative polymerase chain reaction from a clinical virology laboratory in eastern India. Indian Journal of Medical Microbiology.

[ref-24] Smith M (2021). Validating real-time polymerase chain reaction (PCR) assays. Encyclopedia of Virology.

[ref-25] Suviriyapaisal C, Wanachiwanawin D, Limawongpranee S (2017). Detection of *Toxoplasma gondii* by PCR and quantitative PCR with high specificty and lower limit of detection. Southeast Asian Journal of Tropical Medicine and Public Health.

[ref-26] Truong M, Šlapeta J (2023). Analytical sensitivity of a multiplex quantitative PCR for *Toxoplasma gondii* and *Neospora caninum*. Parasitology Research.

[ref-27] Winters AD, Romero R, Graffice E, Gomez-Lopez N, Jung E, Kanninen T, Theis KR (2022). Optimization and validation of two multiplex qPCR assays for the rapid detection of microorganisms commonly invading the amniotic cavity. Journal of Reproductive Immunology.

[ref-28] Xu Y, Lv Y, Lin M, Li M, Cui D, Wang Y, Shen C, Xie J (2024). A novel multiplex real-time PCR assay for the detection of cytomegalovirus, Epstein-Barr virus, herpes simplex virus 1/2 and strategies for application to blood screening. Diagnostic Microbiology and Infectious Disease.

[ref-29] Yamamoto L, Filho AGA, Queiroz JA, De Carvalho MHB, Rodrigues JC, Kanunfre KA, Francisco RPV, Okay TS (2020). Performance of a multiplex nested polymerase chain reaction in detecting 7 pathogens containing DNA in their genomes associated with congenital infections. Archives of Pathology & Laboratory Medicine.

[ref-30] Zhou L, Gong R, Lu X, Zhang Y, Tang J (2015). Development of a multiplex real-time PCR assay for the detection of *Treponema pallidum*, HCV, HIV-1, and HBV. Japanese Journal of Infectious Diseases.

[ref-31] Zimmerman SF, Bonon SHA, Marba STM (2025). Systematic review on molecular detection of congenital and neonatal infections caused by TORCH and SARS-CoV-2 in newborns’ cerebrospinal fluid. Revista Paulista de Pediatria.

