# Supplementary material for: Development and analytical validation of a multiplex qPCR assay for Toxoplasma gondii, Treponema pallidum, cytomegalovirus, and herpes simplex virus types 1 and 2 in neonates
Source: PeerJ. 2026 Sep 15;14:e21695. doi: 10.7717/peerj.21695 (PMC13588099; doi:10.7717/peerj.21695)
Supplement: Supplementary file 1 [file peerj-14-21695-s003.docx]

**Table S1A: Dimerization potential of primers (Self-Dimer & Hetero-Dimer).**

| **Organism** | **Primer Interaction** | **Type** | **ΔG (kcal/mol)** | **Risk** |
| --- | --- | --- | --- | --- |
| *T. gondii* | Forward | Self-dimer | -5.19 | Low |
|  | Reverse | Self-dimer | -7.05 | Low |
|  | Forward + Reverse | Hetero-dimer | -6.57 | Low |
| *T. pallidum* | Forward | Self-dimer | -3.61 | Low |
|  | Reverse | Self-dimer | -6.30 | Low |
|  | Forward + Reverse | Hetero-dimer | -4.88 | Low |
| CMV | Forward | Self-dimer | -3.61 | Low |
|  | Reverse | Self-dimer | -3.54 | Low |
|  | Forward + Reverse | Hetero-dimer | -4.74 | Low |
| HSV-1 | Forward | Self-dimer | -12.90 | High |
|  | Reverse | Self-dimer | -3.61 | Low |
|  | Forward + Reverse | Hetero-dimer | -7.18 | Low |
| HSV-2 | Forward | Self-dimer | -9.28 | High |
|  | Reverse | Self-dimer | -9.49 | High |
|  | Forward + Reverse | Hetero-dimer | -5.12 | Low |

**Table S1B: Dimerization potential of probes (Self-Dimer).**

| **Organism** | **Probe** | **Type** | **ΔG (kcal/mol)** | **Risk** |
| --- | --- | --- | --- | --- |
| *T. gondii* | Probe | Self-dimer | -7.05 | Low |
| *T. pallidum* | Probe | Self-dimer | -9.75 | High |
| CMV | Probe | Self-dimer | -7.05 | Low |
| HSV-1 | Probe | Self-dimer | -5.09 | Low |
| HSV-2 | Probe | Self-dimer | -13.80 | High |

**Table S1C: Hetero-dimerization potential between primers and probes.**

| **Organism** | **Interaction** | **ΔG (kcal/mol)** | **Risk** |
| --- | --- | --- | --- |
| T. gondii | Forward + Probe | -10.09 | High |
|  | Reverse + Probe | -7.05 | Low |
| T. pallidum | Forward + Probe | -6.21 | Low |
|  | Reverse + Probe | -7.74 | Low |
| CMV | Forward + Probe | -4.74 | Low |
|  | Reverse + Probe | -5.50 | Low |
| HSV-1 | Forward + Probe | -8.26 | Low |
|  | Reverse + Probe | -6.69 | Low |
| HSV-2 | Forward + Probe | -7.07 | Low |
|  | Reverse + Probe | -3.61 | Low |
