# Supplementary material for: Development and analytical validation of a multiplex qPCR assay for Toxoplasma gondii, Treponema pallidum, cytomegalovirus, and herpes simplex virus types 1 and 2 in neonates
Source: PeerJ. 2026 Sep 15;14:e21695. doi: 10.7717/peerj.21695 (PMC13588099; doi:10.7717/peerj.21695)
Supplement: Supplementary file 2 [file peerj-14-21695-s004.docx]

**Table S2: Performance of the optimized Multiplex assay**

| **Multiplex Set** | **Target Organism** | **Fluorophore** | **Cq Value** | **Max RFU** |
| --- | --- | --- | --- | --- |
| SET 1 | CMV | FAM | 23.29 | 944 |
|  | HSV-1 | Cy5.5 | 24.52 | 1926 |
|  | HSV-2 | Texas Red | 23.36 | 1099 |
|  | IAC | Cy5 | 26.53 | 589 |
|  | Negative Controls | All Channels | ND | ND |
| SET 2 | *T. gondii* | FAM | 24.06 | 1140 |
|  | *T. pallidum* | Cy5.5 | 24.53 | 1195 |
|  | IAC | Cy5 | 27.11 | 691 |
|  | Negative Controls | All Channels | ND | ND |

ND: Not Detected

IAC (0.2 µM)

CMV (0.9 µM)

HSV-1 (0.2 µM)

HSV-2 (0.2 µM)

Figure S2 (a)	Optimization of SET1 with IAC. The ideal concentration for the CMV (FAM) probe was determined with 0.9 µM, while the HSV-1 (Cy 5.5), HSV-2 (Texas Red) and IAC (Cy 5) probes were held constant at 0.2 µM, yielding a high RFU signal for all three targets

IAC (0.2 µM)

*T. gondii* (0.7 µM) µM(FAM)

*T. pallidum* (0.9 µM)

Figure S2 (b)	Optimization of SET2 with IAC. The ideal concentration for the *T. gondii* (FAM) probe was determined with 0.7 µM, while the *T. pallidum* (Cy 5.5), was determined with 0.9 µM and the IAC (Cy 5) probes were held constant at 0.2 µM, yielding a high RFU signal for all three targets
