# Supplementary material for: Development and analytical validation of a multiplex qPCR assay for Toxoplasma gondii, Treponema pallidum, cytomegalovirus, and herpes simplex virus types 1 and 2 in neonates
Source: PeerJ. 2026 Sep 15;14:e21695. doi: 10.7717/peerj.21695 (PMC13588099; doi:10.7717/peerj.21695)
Supplement: Supplementary file 3 [file peerj-14-21695-s005.docx]

**Table S3: Analytical specificity results against non-target microorganisms.**

| **Category** | **Organism** | **Source / Strain** | **Result** | **IAC Amplification** |
| --- | --- | --- | --- | --- |
| **Gram-Positive Bacteria** | *Staphylococcus epidermidis* | ATCC 12228 | Negative | Positive |
|  | *Streptococcus pneumoniae* | Clinical Isolate | Negative | Positive |
|  | *Staphylococcus aureus* | ATCC 25923 | Negative | Positive |
|  | Methicillin-resistant  *Staphylococcus aureus* (MRSA) | Clinical Isolate | Negative | Positive |
|  | *Enterococcus* species | Clinical Isolate | Negative | Positive |
|  | *Enterococcus faecalis* | Clinical Isolate | Negative | Positive |
|  | *Enterococcus faecium* | Clinical Isolate | Negative | Positive |
|  | *Corynebacterium diphtheriae* | Clinical Isolate | Negative | Positive |
| **Gram-Negative Bacteria** | *Escherichia coli* | ATCC 25922 | Negative | Positive |
|  | *Pseudomonas aeruginosa* | ATCC 27853 | Negative | Positive |
|  | *Haemophilus influenzae* | ATCC 49247 | Negative | Positive |
|  | *Neisseria meningitidis* | ATCC 13090 | Negative | Positive |
|  | *Klebsiella pneumoniae* | Clinical Isolate | Negative | Positive |
|  | *Klebsiella oxytoca* | Clinical Isolate | Negative | Positive |
|  | *Salmonella* Typhi | Clinical Isolate | Negative | Positive |
|  | *Salmonella Paratyphi A* | Clinical Isolate | Negative | Positive |
|  | *Salmonella Paratyphi B* | Clinical Isolate | Negative | Positive |
|  | *Shigella dysenteriae* | Clinical Isolate | Negative | Positive |
|  | *Shigella boydii* | Clinical Isolate | Negative | Positive |
|  | *Shigella flexneri* | Clinical Isolate | Negative | Positive |
|  | *Shigella sonnei* | Clinical Isolate | Negative | Positive |
|  | *Citrobacter freundii* | Clinical Isolate | Negative | Positive |
|  | *Serratia marcescens* | Clinical Isolate | Negative | Positive |
| **Fungi (Yeasts)** | *Candida albicans* | ATCC 64124 & Clinical Isolate | Negative | Positive |
|  | *Candida tropicalis* | Clinical Isolate | Negative | Positive |
|  | *Candida parapsilosis* | Clinical Isolate | Negative | Positive |
|  | *Candida rugosa* | Clinical Isolate | Negative | Positive |
|  | *Candida krusei* | Clinical Isolate | Negative | Positive |
|  | *Candida famata* | Clinical Isolate | Negative | Positive |
|  | *Cryptococcus neoformans* | Clinical Isolate | Negative | Positive |
| **Fungi (Molds)** | *Aspergillus flavus* | Clinical Isolate | Negative | Positive |
|  | *Aspergillus nidulans* | Clinical Isolate | Negative | Positive |
|  | *Aspergillus fumigatus* | Clinical Isolate | Negative | Positive |
|  | *Aspergillus terreus* | Clinical Isolate | Negative | Positive |
|  | *Fusarium proliferatum* | Clinical Isolate | Negative | Positive |
|  | *Penicillium chrysogenum* | Clinical Isolate | Negative | Positive |
| **Viruses** | Hepatitis B Virus (HBV) | Clinical Isolate | Negative | Positive |
|  | Epstein-Barr Virus (EBV) | Clinical Isolate (3) | Negative | Positive |
